# Identification of Proteins Involved in Cell Membrane Permeabilization by Nanosecond Electric Pulses (nsEP)

**DOI:** 10.3390/ijms24119191

**Published:** 2023-05-24

**Authors:** Giedre Silkuniene, Uma M. Mangalanathan, Alessandra Rossi, Peter A. Mollica, Andrei G. Pakhomov, Olga Pakhomova

**Affiliations:** 1 Frank Reidy Research Center for Bioelectrics, Old Dominion University, Norfolk, VA 23508, USA; gsilkuni@odu.edu (G.S.); umamangalanathan@gmail.com (U.M.M.); alessandra.rossi@uniroma1.it (A.R.); apakhomo@odu.edu (A.G.P.); 2 Institute for Digestive System Research, Lithuanian University of Health Sciences, 44307 Kaunas, Lithuania; 3 Department of Translational Medicine, Sapienza University of Rome, 00185 Rome, Italy; 4 College of Health Sciences, Old Dominion University, Norfolk, VA 23508, USA; pmollica@odu.edu

**Keywords:** membrane lesions, membrane repair, membrane proteins, nsPEF, electroporation, electropermeabilization

## Abstract

The study was aimed at identifying endogenous proteins which assist or impede the permeabilized state in the cell membrane disrupted by nsEP (20 or 40 pulses, 300 ns width, 7 kV/cm). We employed a LentiArray CRISPR library to generate knockouts (KOs) of 316 genes encoding for membrane proteins in U937 human monocytes stably expressing Cas9 nuclease. The extent of membrane permeabilization by nsEP was measured by the uptake of Yo-Pro-1 (YP) dye and compared to sham-exposed KOs and control cells transduced with a non-targeting (scrambled) gRNA. Only two KOs, for SCNN1A and CLCA1 genes, showed a statistically significant reduction in YP uptake. The respective proteins could be part of electropermeabilization lesions or increase their lifespan. In contrast, as many as 39 genes were identified as likely hits for the increased YP uptake, meaning that the respective proteins contributed to membrane stability or repair after nsEP. The expression level of eight genes in different types of human cells showed strong correlation (R > 0.9, *p* < 0.02) with their LD_50_ for lethal nsEP treatments, and could potentially be used as a criterion for the selectivity and efficiency of hyperplasia ablations with nsEP.

## 1. Introduction

Electroporation or electropermeabilization refers to a transient or permanent disruption of the membrane barrier function when the transmembrane potential exceeds 200–500 mV [1,2,3,4,5,6]. Diverse applications of electroporation in medicine, biotechnology and research include gene transfer, delivery of chemotherapeutic drugs and vaccines, immune stimulation, and tissue ablation [7,8,9,10].

Electroporation as a physicochemical modification of the membrane has been extensively studied by computational and molecular dynamics (MD) modeling [11,12,13], in artificial lipid bilayers [1,14,15], and in giant unilamellar vesicles [16,17]. Lipid pores formed in these model systems were not thermodynamically stable and resealed within nano- to microseconds once the electric field was removed. More severe electroporation led to the formation of larger pores which were not stable either and caused a nearly instant membrane destruction.

In a stark contrast, the permeabilized state of biomembranes can last from seconds to hours. Long-lasting electropermeabilization was consistently reported in diverse bacterial, plant, and mammalian cells exposed to electric field pulses from nano- to millisecond duration [3,7]. The leaky state of the cell membrane could be revealed minutes after electroporation by the uptake of fluorescent dyes which do not cross the intact membrane, such as propidium and Yo-Pro-1 (YP); by delayed entry of Ca^2+^ and Tl^+^ ions into cells which do not express voltage-gated ion channels; by the continued loss of K^+^ and ATP from the cytosol; by colloid-osmotic swelling and eventual cell rupture; and by measuring membrane electrical resistance. It is not known how the longevity of the permeabilized state depends on the membrane composition or the electroporation protocol, except for a general notion that more severe damages take longer to reseal. Moreover, the repair time of the electroporated membrane in living cells depends on the activation of the specialized cellular machinery that patches or removes lesions.

The discrepancy between the models and observations in living cells suggested that electroporation of biomembranes is a complex process which may involve membrane proteins [3,6,18], cytoskeletal scaffold, cholesterol, and oxidation of membrane lipids. However, experimental data remain controversial and do not prove any of the above mechanisms as a unique or prevalent cause of the long-lasting permeabilization.

Membrane proteins are attracting increasing interest as potential targets of electroporation, especially with ultra-short electric pulses whose intensity may be strong enough to cause conformational changes. Such changes may lead to function loss and even to pore formation within the protein itself. Highly charged protein domains, such as the voltage sensor subunit of voltage-gated ion channels, can be particularly vulnerable to supraphysiological voltages. The electric field may have an effect similar to certain mutations which cause the leakage of cations through the gating pore (so called “omega” current) [19,20,21]. Indeed, MD modeling demonstrated the possibility of pore formation in the voltage sensors of voltage-gated ion channels [22,23], and these pores could expand into so-called complex pores with lifetimes much longer than in conventional lipid pores. Another MD study showed a trans-protein pore formation in the human aquaporin channel, but it closed within about 20 ns after turning the electric field off [24]. While the MD findings of protein pore opening have yet to be confirmed by wet lab experiments, long-lasting changes in the conductance and voltage response of endogenous ion channels caused by intense nanosecond electric pulses (nsEP) were demonstrated by independent groups using patch clamp [25,26,27,28]. Nanopores created by high-voltage nsEP in living cells displayed complex conductive properties (voltage sensitivity, inward rectification, and ion selectivity [27,29,30,31]), suggesting that these pores could be protein structures rather than simple lipid pores. Our previous discussion of these properties [29,30] has leaned towards explaining them a funnel-like shape of lipid nanopores, but the protein pore formation was not ruled out. Finally, the expression of voltage-gated calcium channels augmented cell membrane susceptibility to nsEP, suggesting that their presence assisted pore formation or increased pore lifetime [32].

The great variety of membrane proteins makes it difficult to single out those which should be tested for the electric field sensitivity by MD or in direct experiments. To assist this selection, we performed a high-throughput screening of proteins contributing to electroporation, by creating CRISPR knock-outs (KOs) of 316 genes which encode membrane proteins, and testing whether each mutation changed cell permeabilization by nsEP. We looked primarily for the KOs which reduced the electroporative dye uptake, suggesting that a transmembrane pore could have opened in the eliminated protein, or the protein assisted electropermeabilization by some other mechanism. Concurrently, we also identified the KOs with increased YP uptake after nsEP treatments. Such KOs identify proteins which aid membrane resilience or assist lesion repairs.

## 2. Results

### 2.1. Dose-Response Dependence for Cell Membrane Permeabilization by 7 kV/cm, 300-ns Pulses

**Figure 1:**
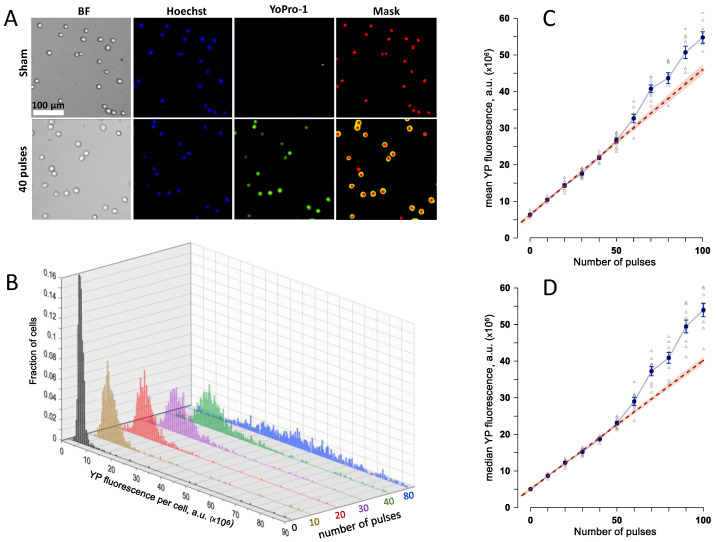
Quantification of cell membrane electropermeabilization by the uptake of Yo-Pro-1 (YP) dye. (**A**) Sample images of U937 cells after a sham exposure (control) and after a train of 40, 300-ns pulses at 7 kV/cm. BF, bright field; Hoechst, cell nuclei stained with Hoechst dye; Yo-Pro-1, dye fluorescence in electroporated cells; Mask, automated labeling of the nucleus and cell regions for measuring the integral YP fluorescence per cell. (**B**) The effect of the number of 300-ns, 7 kV/cm pulses on the frequency distribution of YP fluorescence in exposed cells. (**C**,**D**): The mean (**C**) and median (**D**) values of YP emission per cell, averaged across 4–7 independent experiments, for electroporation with different numbers of 300-ns, 7 kV/cm pulses. Error bars are the standard error. Dashed line is the best linear fit for the data between 0 and 50 pulses; the shaded area along the fit line is the 95% confidence interval. See text for more detail.

These starting experiments were aimed at identifying nsEP exposure parameters optimal for the detection of either increase or decrease of YP uptake due to gene KOs. U937-Cas9 cell culture at 2–4 × 10^4^ cells/mL was supplemented with Hoechst and YP dyes and dispensed into 1-mm gap electroporation cuvettes, at 100 µL per cuvette. The cuvettes were exposed to different numbers of 300-ns pulses, from zero (sham exposure) to 100. Pulse voltage and repetition rate were kept constant at 700 V and 20 Hz. In 50 min, YP uptake by individual cells was measured by fluorescence imaging (Figure 1A). Cell-permeable Hoechst dye labeled nuclei of all cells, to assist automated cell identification and YP fluorescence measurements. Exposures to 10–50 nsEP increased YP uptake linearly, consistently with earlier studies [33], with a bell-shaped distribution across the cell population (Figure 1B). Further increase in pulse number scattered the YP distribution across a wide range and its peak became poorly defined. Both the mean and the median fluorescence values diverged from the linear dependence and showed much higher fluctuations than at lower nsEP doses (Figure 1B–D). The likely cause for such changes was the emergence of secondary effects downstream of the membrane permeabilization, such as cell swelling and membrane rupture [29,34]. Such effects could obscure changes in membrane permeabilization in gene KOs, and had to be avoided. For most of the subsequent experiments, we chose a dose of 20 pulses, which enabled quantitation of both up- and downward YP uptake changes.

### 2.2. Validation of CRISPR-Cas9 Genome Editing

**Figure 2:**
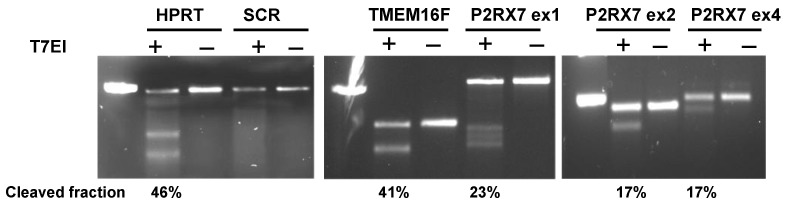
Genomic cleavage detection in U937-Cas9 cells. Cells were transduced with gRNA targeting *HPRT* and *TMEM16F* genes, as well as *P2RX7* gene at three different exons. Negative control cells were treated with a non-targeting gRNA (SCR). The targeted DNA sites were amplified by PCR, followed by a cleavage assay with T7EI endonuclease (T7EI+) or without it (T7EI−), and visualized in a 2% agarose gel using a UV transilluminator. The left column in each gel is a 500 bp marker. See text for more details.

Targeted gene editing by CRISPR was validated in several selected KOs by a genomic cleavage detection assay. Target regions were amplified by PCR, to create heteroduplex DNA which was cut by a mismatch-specific endonuclease T7EI. Figure 2 illustrates the detection of cleaved DNA fragments by gel electrophoresis in KOs of hypoxantine phosphoribosyltransferase (*HPRT*), anoctamin 6 (*TMEM16F*), and purinergic receptor P2X7 (*P2RX7*) genes, but not in a sample transduced with scrambled gRNA (SCR, non-targeting gRNA). Cleaved segments were present in samples treated with the endonuclease (T7EI+ wells) but not in the negative controls (T7EI− wells). *P2RX7* KOs were generated by a mixture of gRNAs targeting three different exons, which were amplified using respective primers and tested separately. The cleaved fraction reached close to 50%, which is the theoretical detection limit for this method.

### 2.3. Effect of Gene KOs on YP Uptake by Electropermeabilized Cells

In the first set of experiments, we generated 15–20 gene KOs and 2–4 SCR controls, to be treated with nsEP and analyzed concurrently. Each of the variants was split into two samples, one of which was electroporated (20, 300-ns pulses at 7 kV/cm) and the other one was sham-exposed. YP uptake was determined in 50 min after the nsEP or sham treatment. The median and mean YP fluorescence values per cell were calculated from the analysis of 400–2000 cells per sample. The subtraction of the respective values measured in the matching sham-exposed sample corrected the result for the “spontaneous” YP uptake, yielding the YP fluorescence change solely due to the nsEP treatment. The experiments were repeated at least 3 times, on different days. The averaged mean and median values of YP uptake per cell were compared to the parallel SCR controls. Possible hits were determined by Dunnet’s test at *p* < 0.05 and the strictly standardized mean difference (SSMD) [35,36] values exceeding 1.0 (or smaller than −1.0 for reduced YP uptake).

**Figure 3:**
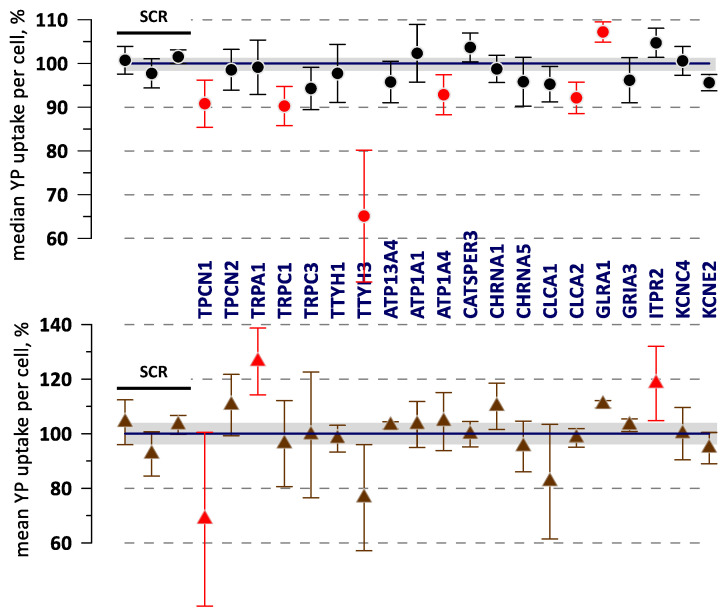
Data from a sample experiment comparing electroporation-induced YP uptake in different gene KO variants (labeled) and in control variants transduced with the non-targeting gRNA (“SCR”, the first three datapoints). YP uptake was measured 50 min after electroporation by a train of 20, 300-ns, 7 kV/cm pulses and plotted as a median (top graph) and mean YP fluorescence per cell. Error bars are the standard error from three independent experiments. Shaded areas are the standard error across the control samples. Datapoints with SSMD > 1 or SSMD < −1 are marked by red symbols. None of the KOs in this group showed statistically significant differences from the control using Dunnet’s test.

A sample experiment that compared 20 KOs with 3 SCR controls is illustrated in Figure 3. For the most part, mean and median values followed the same trends, but changes in the median values and their variability typically were smaller. The SSMD calculated for mean and median values could exceed 1.0 (or −1.0) threshold for different KOs, and all of them were considered as possible hits. However, none of the KO data in Figure 3 reached a statistically significant difference from the control using Dunnet’s test.

**Figure 4:**
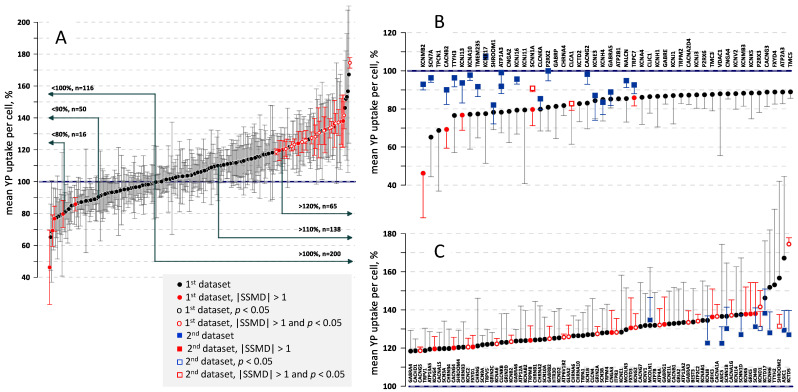
The effect of gene knock-outs on cell membrane permeabilization, as measured by YP dye entry following nsEP exposure. Graph show the mean value of YP uptake per cell (+/− s.e.) in % to control cells transduced with a scrambled gRNA. Datapoints marked by red color identify changes with SSMD > 1.0 or <−1.0. Open symbols mark statistically significant differences from the control at *p* < 0.05. (**A**): A total of 316 knockouts were sorted based on the YP uptake. Labels show the number of knockouts with increased and decreased YP uptake, in 10% steps. Note about a 3-fold larger number of knockouts which displayed the increased YP uptake. (**B**,**C**): The end regions of the graph from (**A**) with gene names added. The panels also show the data from replication sets of experiments (square symbols). See text for more details.

The mean and median data from these and other similar experiments, covering a total of 316 tested KOs, are summarized in the Supplementary Table S1 and in Figure 4A. In the table, the genes are sorted in the alphabetical order, and the averages of the mean and median values for each gene are presented along with the respective SSMD and *t*-values using Dunnet’s test (*t* > 3.0 corresponds to *p* < 0.05).

In Figure 4A, gene KOs are sorted from the smallest to the largest mean YP uptake. The highlight of this graph is its profound asymmetry with respect to the 100% midpoint. The number of KOs with the mean YP uptake above 110% and 120% was 135 and 65, respectively, versus only 50 below 90% and 16 below 80%. The median YP uptake showed a similar unequal distribution, with a 2- to 4-fold larger number of KOs with the increased YP uptake (e.g., 58 KOs > 110% but only 15 KOs < 90%). The predominance of the YP increase trend is emphasized by the fact that SSMD exceeded 1.0 in as many as 28 KOs, but fell below −1.0 in only 5 KOs (red symbols in Figure 4A). Likewise, the difference from SCR control was statistically significant at *p* < 0.05 in 17 KOs with increased YP uptake (open symbols), but not in a single KO with a decreased YP uptake. Such response pattern suggests that KO of many different genes can make cell membrane more vulnerable to nsEP, by altering its resilience or repair, while KOs which reduce membrane permeabilization by nsEP are only a few or non-existing.

The regions of Figure 4A curve with the lowest and the highest YP uptake are expanded and the KOs are labeled in panels B and C of Figure 4. While the statistical analyses pointed to the potential hits, the only way to prove these findings was their replication in independent series of experiments. The data from two replication series are presented by square symbols in Figure 4B,C.

The first replication series was focused on gene KOs with YP uptake lower than in SCR controls. The KOs were generated de-novo and tested in 4 independent experiments within a shorter time frame after the transduction (see Section 4 for more detail). In addition, nsEP exposure was changed from 20 pulses to 40 pulses, to allow for a better detection of the expected weakening of effects (see Figure 1B). Similar to the first series of experiments, most KOs were not different from the SCR control (Figure 4B). However, two of them, α subunit of amiloride-sensitive sodium channel (*SCNN1A*) and regulator 1 for calcium-activated chloride channel (*CLCA1*), showed a statistically significant reduction in YP uptake and SSMD < −1. The hit for *SCNN1A* replicated the observation of SSMD < −1 from the previous set of experiments, making this gene the most likely hit in the reduced YP uptake group. Several other gene KOs, although did not reach the statistical significance, showed a good match between the data collected in the two independent sets of experiments. Specifically, shroom family member 1 (*SHROOM1*), voltage-gated (VG) chloride channel Ka (*CLCNKA*), β subunit of VG potassium channel (*KCNE3*), α subunit of VG potassium channel (*KCNH4*), α5 subunit of GABA(A) receptor (*GABRA5*), and transient receptor protein 7 (*TRPC7*) may be considered as potential hits for future studies. The analysis of the median YP uptake data did not reveal any additional potential hits.

The second replication series was focused on gene KOs with YP uptake higher than in SCR controls (Figure 4C). With many potential hits identified in the first set of experiments, we focused on statistical power of the replication rather than on the inclusion of all the suspects. The KOs were generated de-novo and tested in as many as 8 independent experiments, using the same nsEP parameters as previously (20 pulses, 300 ns, 7 kV/cm). While the differences from the control (square symbols) were smaller than observed in the previous series, it is worth noting that a higher YP uptake, to a minimum of 120%, was confirmed in all the KOs tested. A KO for alpha subunit of VG potassium channel (*KCND1*) displayed a statistically significant increase to 130–140% in both sets of experiments, making it the most likely hit. Shroom family member 2 (*SHROOM2*) and tetramerization domain for potassium channel (*KCTD5*) KOs showed a significant effect in one of the series and a strong, albeit not significant increase in the other series. On the total, as many as 35 KOs displayed a significant (*p* < 0.05) increase in the mean and/or median YP uptake, along with SSMD > 1.0, in one or both series of experiments; 4 more KOs were added to this list of possible hits based on the reproducibility changes seen in the two series of experiments.

**Figure 5:**
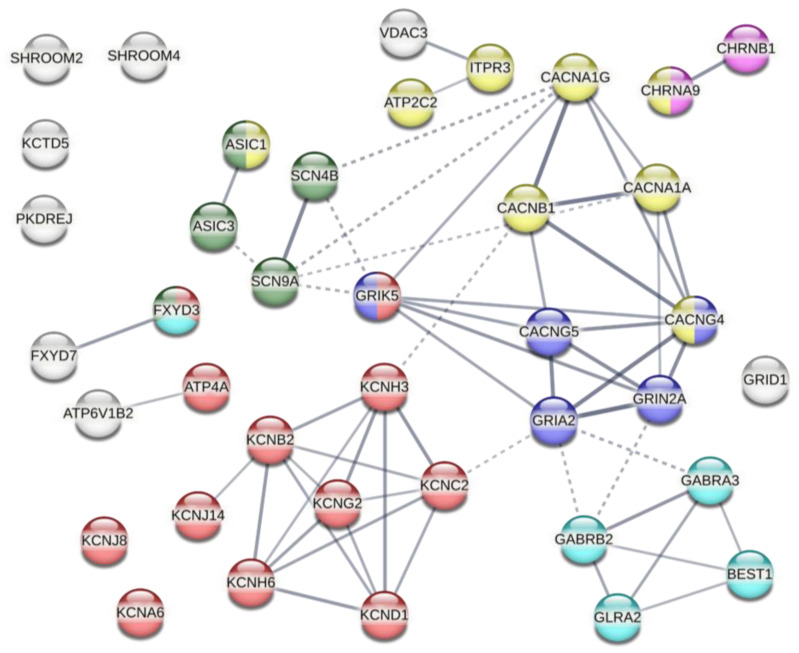
The families of genes whose knock-outs led to a significant increase in YP uptake following electroporation with nsEP. The protein–protein interactions were categorized by STRING database v. 11.5 at the confidence level of 0.5. Colors identify proteins related to Ca^2+^ (yellow), K^+^ (red), Na^+^ (green), or Cl^−^ transport (cyan). Blue and magenta colors are for proteins related to the glutamate and acetylcholine receptor complexes, respectively.

These 39 genes were analyzed using STRING database v. 11.5. and presented in Figure 5 as several functionally or structurally related groups. The major identified groups were the genes coding for proteins which participate in K^+^ and Ca^2+^ handling, followed by those related to Na^+^ and Cl^−^ channels, glutamate and acetylcholine receptor complexes; still other genes had no identified role or connections with other genes in the group (or the information is too scarce and not in the database). While we could not single out any specific genes uniquely responsible for the higher YP uptake, it makes sense that KOs of many genes, belonging to diverse groups, reduce cell membrane stability and repair capacity after the electric insult. It is also possible that group includes some false positives, which cannot be avoided in wide-range screens and should be validated individually in subsequent studies.

### 2.4. The Correlation of Gene Expression with Cell Survival

**Figure 6:**
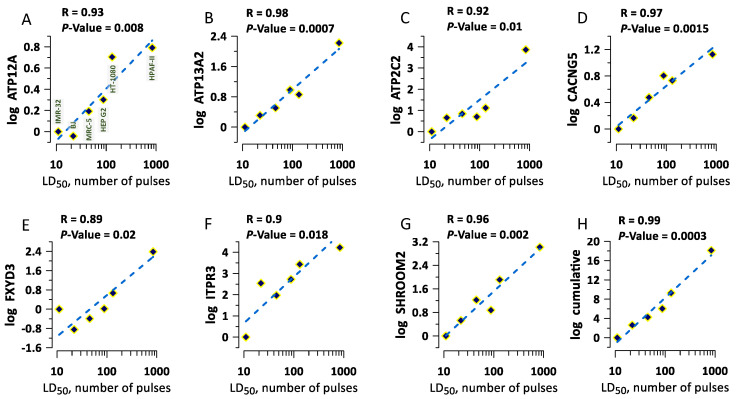
Selected gene expression levels correlate with sensitivity to lethal nsEP treatments. The sensitivity (LD_50_) data are from a previous study in 6 different human cell lines [39]. Panels (**A**–**G**) show the difference in the expression of a particular gene (labeled at the Y axis). The expression level in the most sensitive cell type (IMR-32) was taken as a baseline (zero). The names of the cell lines are labeled in panel (**A**). The datapoints were fit with a linear function (dashed lines). Labels above the plots are the correlation coefficient (R) and the statistical significance of correlation (*p*-value). Panel (**H**) shows the cumulative expression data, pooled together from plots in (**A**–**G**). See text for more details.

An increased electroporative YP uptake in a gene KO means that the missing or altered protein helped the membrane stability or repair in the wild-type variant. Then, one can expect that at least some of these proteins contribute to cell survival after nsEP treatments, such as tissue and tumor ablation. The sensitivity of different types of cells to nsEP depends on specific pulse parameters and varies in very wide ranges [37,38,39]. For example, the lethal dose of 300-ns pulses that killed 50% of cells (LD_50_) in six different human cell types differed as much as 80-fold [39]. However, cellular mechanisms underlying the different sensitivity remain unclear. Using the Genevestigator database [40], we extracted the expression levels of 60 genes whose KOs resulted in higher YP uptake, and compared them across the six cell types with known LD_50_ [39]. When the difference in the expression levels of an individual gene was plotted against the respective LD_50_, we found a strong (R ≥ 0.9) and highly significant (*p* ≤ 0.02) correlation for eight of the checked gens (Figure 6A–G). Pooling together the expression levels for these genes yields a nearly perfect correlation with LD_50_ (R = 0.986, *p* = 0.0003, Figure 6H). We infer that comparing the expression levels of these genes could be a method to predict the sensitivity of different cell types to nsEP, as well as the selectivity and efficiency of nsEP ablation treatments.

## 3. Discussion

This is the first study that attempted to identify genes which encode for proteins affecting cell membrane permeabilization by nsEP, by either inhibiting or facilitating the electroporative dye uptake into cells. One of the hypotheses tested was that the transmembrane passageways created by electroporation could be within membrane protein structures or stabilized by membrane proteins. A knock-out of such proteins would lead to a reduction in the electroporative dye entry. We identified two likely candidates (*SCNN1A* and *CLCA1*) for this role, which needs to be investigated and confirmed by other methods. *SCNN1A* encodes alpha subunit of the epithelial sodium channel (ENaC) which mediates sodium and fluid transport [41]. *CLCA1* encodes for a regulator protein involved in mediating calcium-activated chloride conductance [42,43]. We have not identified any structural similarity or functional connection between these proteins.

Concurrently, we identified tens of genes whose KOs increased the electroporative dye uptake, i.e., reduced the cell resilience to damage by nsEP. These genes must be encoding proteins that contribute to membrane stability or repair in wild-type variants, and it makes sense that such proteins are numerous and may belong to diverse groups. We further demonstrated that the level of expression of some of these proteins correlates with cell survival following lethal nsEP insults. This knowledge could assist the identification of the exact cell mechanisms responsible for nsEP sensitivity, and can be employed to predict the outcome of nsEP ablation treatments for cancer and other hyperplasia.

With many genes tested and many potential hits identified, it is important to note that the CRISPR-Cas9 approach employed in our study does not guarantee that all relevant genes will be revealed by a change in YP uptake. CRISPR-Cas9 produces double-strand breaks in the target gene, which are repaired by the error-prone non-homologous end joining. The resulting insertions and deletions may or may not lead to a frameshift and the loss of the protein function. CRISPR-Cas9 produces a heterogenous population of cells with various genetic alterations [44], where the response of the cell fraction with a changed sensitivity to nsEP can be masked. It can also be concealed by the overexpression of genes with a similar function (paralogs) [45]. Over time, the fraction of “true” KOs in the heterogenous population may decrease (because cells with no or less severe genetic alterations may proliferate faster), and the suspected effect seen in experiments done early after transduction may disappear later. This was the case, for example, for alpha subunit of the sodium/potassium ATPase (ATP1A1) gene, whose KO showed an initially strong difference which disappeared later, resulting in a high variance and the lack of statistical significance, so ATP1A1 gene did not make it to the list of potential hits. Nonetheless, the data prompted additional studies using shRNA, which confirmed the contribution of this gene to membrane damage by nsEP (to be reported in a separate paper).

Cloning each of the CRISPR-Cas9 mutants could potentially address some of the above issues, but would be a prohibitively extensive and time-consuming effort. Therefore, we opted for a protocol more prone to false negatives (=missing relevant genes) in favor of identifying at least some of the real targets. The profound asymmetry of the frequency of up- and downward changes in YP uptake (Figure 4A), the replication of changes in independent experiments (Figure 4B,C), and the correlation of gene expression with cell survival (Figure 6) provide strong evidence that some (or many) of the identified genes are true targets rather than statistical false positives. Our data provide the basis for more detailed studies of the cellular mechanisms which govern membrane damage, repair, and cell survival after an electric insult.

## 4. Materials and Methods

### 4.1. Reagents and Materials

RPMI-1640 medium with L-glutamine and 25 mM HEPES was purchased from Corning (Corning, NY, USA). Antibiotics penicillin and streptomycin were obtained from Gibco (Gaithersburg, MD, USA). Fetal bovine serum (FBS) was from Atlanta Biologicals (Lawrenceville, GA, USA). All other reagents, including a fluorescent nuclear staining dye Hoechst 33342 (Hoechst), a membrane permeabilization indicator Yo-Pro-1 (YP), LentiArray Ion Channel library (catalog No. A42277), positive and negative controls lentiviruses, GeneArt Genomic Cleavage Detection Kit, TMEM16F gRNA and a Cas9 lentivirus were purchased from Thermo Fisher Scientific (Waltham, MA, USA).

### 4.2. Cell Culture and Gene Knock-Out Procedures

Experiments were performed in U937 human monocytes, a suspension cell line derived from a histiocytic lymphoma (ATCC, Manassas, VA, USA). Cell culture was maintained in a 5% CO_2_ incubator at 37 °C in the RPMI-1640 medium supplemented with 10% FBS, 100 IU/mL penicillin, and 0.1 mg/mL streptomycin.

Cells stably expressing Cas9 nuclease (U937-Cas9) after transduction with a LentiArray Cas9 lentivirus were isolated by selection with 8 µg/mL blasticidin added to the medium. Ten clones were isolated by limited dilution, expanded, and Cas9 efficiency was determined with a GeneArt Genomic Cleavage Detection Kit. The clone with the highest nuclease efficiency was selected for subsequent experiments to create gene knock-outs (KOs) using CRISPR method.

We used a LentiArray Ion Channel CRISPR library to knock-out over 300 human genes coding for ion channels, pumps, carriers, and proteins with yet unclear function. Gene-specific guiding RNAs (gRNAs) are packed into lentiviruses and arrayed in 96-well plates, and each well contains four gRNAs designed to recognize different regions of the same gene. One gene, TMEM16F, was of interest to us because of the previous work [46] but was not in the library; it was added and processed the same way as the library genes. Non-targeting scrambled (SCR) gRNA packed in lentivirus was used to generate control cells, which were treated the same way as the KOs.

We performed several series of experiments aimed at identifying genes whose KO impact cell membrane permeabilization by electroporation. In the primary series with 316 KOs, U937-Cas9 cells were transduced at the multiplicity of infection of 1 and propagated in the growth medium with 0.5 µg/mL puromycin. Aliquots of KO and SCR cells were diluted at about 10^4^ cells/mL into a fresh medium without puromycin 1–2 days before electroporation experiments. A total of 3 or 4 independent experiments with each of the KOs were performed within a month and a half after the transduction.

A number of KOs with suspected differences in permeabilization compared to SCR controls (see Section 2 for details) were selected for the additional (validation) series of experiments. KO and SCR cells were generated de novo using the same protocol. All electroporation experiments in the validation series were performed during the third week after the transduction, to minimize possible variability due to culture aging.

### 4.3. Genomic Cleavage Detection

CRISPR-Cas9 genome editing efficiency was assessed with the GeneArt Genomic Cleavage Detection Kit (ThermoFisher, Waltham, MA, USA), following the methodology suggested by the manufacturer. The kit is intended to detect indels in the target DNA sequence by agarose gel electrophoresis. It works by amplifying the target region using PCR, creating a heteroduplex DNA by denaturing and re-annealing the amplicons, and treating the heteroduplex with a mismatch-specific T7EI endonuclease. The PCR primers were designed using the Primer-BLAST tool (http://www.ncbi.nlm.nih.gov/tools/primer-blast, accessed on 10 August 2021). The samples were run on 2% agarose gels. Negative controls included DNA samples not incubated with T7EI, as well as cells transduced with SCR gRNA. We also used positive control cells, transduced with gRNA targeting the hypoxanthine phosphoribosyl transferase (HPRT) gene and respective PCR primers acquired from ThermoFisher. Band intensities on agarose gels were analyzed with GelDoc XR+ software (Image Lab TM version 2.0.1.) (BioRad, New York, NY, USA) and the cleaved fraction (CF) was calculated as CF = (B + C)/(A + B + C) where A, B, C are intensities of parental band (A) and cleaved bands (B,C). The maximum detection of mismatched re-annealing by this method is limited to 50% because half of the DNA strands will reanneal with complementary strands that have no indel or the same indel.

### 4.4. Electroporation and Detection of Cell Membrane Permeabilization

In a typical experiment, we electroporated 15–20 gene KO cell variants concurrently with 2–4 SCR controls. Cell suspensions grown to 2–4 × 10^4^ cells/mL were supplemented with fluorescent dyes, 1 μg/mL Hoechst and 1 μM YP. Cell-permeable Hoechst dye labeled nuclei of all cells, to assist cell identification in automated image analyses after electroporation experiments. YP has limited permeability into intact cells and was used as a semi-quantitative marker of plasma membrane permeabilization by electroporation. Two 100-µL aliquots of each cell culture were transferred into a pair of 1-mm gap electroporation cuvettes (BioSmith, Vandergrift, PA, USA). One of the cuvettes in each pair was exposed to nsEP from an AVTECH AVOZ-D2-B-ODA generator (AVTECH Electrosystems, Ottawa, ON, Canada) as described previously [46]. The other cuvette served as a sham-exposed matched control and was subjected to all the same manipulations excluding nsEP exposure. Unless otherwise noted, cells were exposed to a 20-Hz train of 20 pulses at 700 V (7 kV/cm). The pulse amplitude and shape were monitored using a TDS 3052 oscilloscope (Tektronix, Inc., Beaverton, OR, USA). Maximum heating (disregarding heat dissipation) from nsEP was calculated using the adiabatic heat equation [47] and did not exceed 1 and 2 °C for treatments with 20 and 40 pulses, respectively.

Immediately following nsEP exposure, the exposed and the respective sham-exposed sample were diluted by adding 200 µL of the growth medium to the cuvette. The samples were mixed gently, and 200 µL from each sample was transferred to a black-wall 96-well glass-bottom plate; the rest of the suspension was discarded. The 96-well plate was kept at the room temperature on the bench until nsEP exposures were completed for all samples in the group, after which the plate was placed on the microscope stage. Image acquisition began 50 min after the first nsEP treatment, to allow for YP entry into electroporated cells, and for cells to settle onto the bottom of the well. The 50-min interval between nsEP exposure and image acquisition was maintained for all samples in the group.

Images were taken with an inverted Olympus IX83 microscope (Olympus America, Hamden, CT, USA) custom configured with an automated MS-2000 scanning stage (ASI, Eugene, OR, USA), X-Cite 110LED illuminator (Excelitas Technologies Corporation, Waltham, MA, USA) and an ORCA-Flash4 sCMOS camera (Hamamatsu, Japan). To catch a high-resolution image of the bottom of each well, 9 images of adjacent regions were taken with a 20×, 0.45 NA objective and stitched together. Hoechst and YP fluorescence images were taken with DAPI and FITC filter cubes (ex/em 350/460 nm and 480/535 nm, respectively). All image acquisition steps, including the cube selection, stage re-positioning, and synchronization with illumination and camera operation, were accomplished automatically with CellSens software, version 3.2 (Olympus).

### 4.5. Image Analysis, Quantification of Membrane Permeabilization, and Hit Selection

Images were analyzed with the Advanced CellScoring package of MetaMorph (Version 7.10.2.240, Molecular Devices, San Jose, CA, USA). A total of 400–2000 cells were automatically identified in each image as Hoechst-positive objects, and YP intensity was measured in each cell. Automated measurements were validated by visual inspection, to exclude possible artifacts. Numerical data were processed with Microsoft Excel (Microsoft, Redmond, WA, USA) and Grapher 16 (Golden Software, Golden, CO, USA).

YP emission intensity was averaged across all cells identified in each sample. The principal metric that was employed to characterize membrane electropermeabilization was the difference in YP emission between the exposed and the matching sham-exposed sample (ΔF). For all samples processed as a group and imaged in one plate (15–20 gene KO samples and 2–4 SCR samples, see Section 4.4 above), all ΔF values were normalized to the mean ΔF of SCR samples, which was taken as 100%. Normalization minimized the impact of variability between individual plates [36]. Normalized ΔF values from 3–8 independent experiments were pooled together to calculate their mean ($\overset{-}{\Delta F}$) and standard error for each of KOs.

The statistical significance of differences in $\overset{-}{\Delta F}$ between KO and SCR cells was calculated by Dunnett’s test for comparison of multiple groups with a single control [48]. The magnitude of suspected effects was also characterized by the strictly standardized mean difference (SSMD), the criterion which is commonly used in high-throughput screening [35] to identify the potential hits [35]. SSMD was calculated as suggested by Zhang [36] for screens with unpaired replicates with unequal variance:

(1) $$SSMD=\frac{{\overset{-}{\Delta F}}_{KO}-{\overset{-}{\Delta F}}_{SCR}}{\sqrt{S_{KO}^{2}+S_{SCR}^{2}}}$$

where ${\overset{-}{\Delta F}}_{KO}$ and $S_{KO}^{2}$ are the mean and its variance for a particular gene KO, and ${\overset{-}{\Delta F}}_{SCR}$ and $S_{SCR}^{2}$ are the respective values for control cells transduced with non-targeting gRNA. With the normalization method described above, ${\overset{-}{\Delta F}}_{SCR}$ equaled 100%. While the interpretation of SSMD varies widely across different studies, the values < 0.5, >0.5, and >1.0 are commonly regarded as weak or no effects, moderate effects, and strong effects, respectively. In this study, we adopted a conservative interpretation and only considered SSMD values > 1 (or <−1 for the responses smaller than controls).

The above calculations were repeated identically but starting with the median of YP fluorescence instead of its mean. Median values are less affected by possible deviations of YP uptake from the bell-shaped distribution (Figure 1B) and spontaneous changes in the small number of highly fluorescent cells, such as dead cells randomly present in samples.

### 4.6. Gene Expression Analysis in Cell Lines

Gene expression data for selected genes were obtained from the Genevestigator database [40] for six human cell lines (pancreatic adenocarcinoma HPAF-II, hepatocellular carcinoma Hep-G2, fibrosarcoma HT-1080, neuroblastoma IMR-32, and fibroblasts MRC-5 and BJ). These cell lines were used in the previous study of cell killing efficiency by nsEP and displayed LD_50_ differing as much as 80-fold [39]. The expression data for the selected cell lines were collected using the Affymetrix Human Genome U133 Plus 2.0 Array platform. Only control samples (no experimental treatment) were included in the gene expression analysis.

## Data Availability

The data presented in this study are available on request from the corresponding author.

## Supplementary Materials

The following supporting information can be downloaded at: https://www.mdpi.com/article/10.3390/ijms24119191/s1.
