# Supplementary material for: Identification of Proteins Involved in Cell Membrane Permeabilization by Nanosecond Electric Pulses (nsEP)
Source: Int J Mol Sci. 2023 May 24;24(11):9191. doi: 10.3390/ijms24119191 (PMC10253066; doi:10.3390/ijms24119191)
Supplement: Supplementary file 1 [file ijms-24-09191-s001.zip › ijms-2397044-supplementary.pdf]

**Table S1**. Effect of different gene knock-outs on YO-PRO-1 dye uptake by electroporated cells. The data for tested genes are presented in the alphabetical order. The average values of the mean or median YP fluorescence per cell are express in % to the parallel control (data not shown). The differences from the parallel control are expressed by t-values; the value over 3.0 corresponds to p<0.05 using Dunnet's test for the comparison of multiple groups with control. See text for more details.

| | | MEDIAN | | | | | MEAN | | | |
|---|---|---|---|---|---|---|---|---|---|---|
| gene name | | Average, % | s.e., % | t-value | SSMD | | Average, % | s.e., % | t-value | SSMD |
| | | | | | | | | | | |
| ANO6 | | 104.8 | 1.8 | 1.87 | 0.66 | | 101.1 | 7.05 | 0.09 | 0.03 |
| ASIC1 | | 112.9 | 7.75 | 1.64 | 0.8 | | 167.2 | 43.05 | 1.55 | 0.77 |
| ASIC2 | | 102.1 | 12.98 | 0.16 | 0.09 | | 92.5 | 10.89 | -0.55 | -0.22 |
| ASIC3 | | 114 | 3.17 | 3.75 | 1.6 | | 116.6 | 9.24 | 1.64 | 0.77 |
| ASIC4 | | 99.3 | 9.75 | -0.07 | -0.03 | | 97 | 8.22 | -0.26 | -0.09 |
| ASIC5 | | 107.7 | 7.24 | 1.05 | 0.51 | | 136.5 | 37.9 | 0.96 | 0.47 |
| ATP12A | | 107.9 | 2.28 | 2.62 | 1 | | 123.8 | 18.22 | 1.27 | 0.68 |
| ATP13A1 | | 103.5 | 6.3 | 0.5 | 0.23 | | 96.2 | 7.52 | -0.34 | -0.12 |
| ATP13A2 | | 107.6 | 5.45 | 1.31 | 0.65 | | 133.4 | 21.06 | 1.56 | 0.85 |
| ATP13A4 | | 95.8 | 4.74 | -0.85 | -0.39 | | 103 | 1.37 | 0.72 | 0.22 |
| ATP13A5 | | 102.8 | 2.13 | 1.13 | 0.46 | | 119.4 | 9.15 | 1.86 | 0.77 |
| ATP1A1 | | 102.3 | 6.59 | 0.34 | 0.18 | | 103.4 | 8.41 | 0.36 | 0.17 |
| ATP1A2 | | 105.8 | 8.06 | 0.71 | 0.35 | | 116.5 | 16.17 | 0.98 | 0.45 |
| ATP1A3 | | 78 | 8.48 | -2.57 | -1.26 | | 78.3 | 10.85 | -1.82 | -0.79 |
| ATP1A4 | | 92.9 | 4.57 | -1.48 | -0.67 | | 104.5 | 10.63 | 0.39 | 0.18 |
| ATP2A1 | | 105.7 | 7.62 | 0.74 | 0.37 | | 101.6 | 3.91 | 0.23 | 0.07 |
| ATP2A2 | | 101.5 | 0.97 | 0.68 | 0.21 | | 105.3 | 5.24 | 0.8 | 0.33 |
| ATP2A3 | | 102.8 | 14.57 | 0.19 | 0.1 | | 88.9 | 6.16 | -1.09 | -0.37 |
| ATP2B1 | | 91.4 | 1.66 | -4.58 | -1.92 | | 85.3 | 1.02 | -2.12 | -0.62 |
| ATP2B2 | | 98.5 | 4.14 | -0.36 | -0.17 | | 101 | 7.69 | 0.1 | 0.04 |
| ATP2C1 | | 105.7 | 6.09 | 0.93 | 0.45 | | 93.1 | 5.85 | -0.83 | -0.29 |
| ATP2C2 | | 109.9 | 2.05 | 3.93 | 1.2 | | 134.1 | 3.6 | 5.9 | 1.64 |
| ATP4A | | 115.5 | 4.8 | 3.09 | 1.05 | | 132 | 8.65 | 3.28 | 1.05 |
| ATP5B | | 92.1 | 5.17 | -1.5 | -0.82 | | 89.1 | 13.57 | -0.73 | -0.35 |
| ATP6V1A | | 96.6 | 1.38 | -2.11 | -0.85 | | 103.2 | 5 | 0.38 | 0.13 |
| ATP6V1B2 | | 106 | 2.71 | 1.79 | 0.73 | | 125.6 | 1.65 | 5.74 | 1.75 |
| ATP7A | | 95.7 | 2.02 | -1.96 | -0.86 | | 104.5 | 13.17 | 0.3 | 0.13 |
| ATP7B | | 110.8 | 6.29 | 1.64 | 0.84 | | 131.9 | 17.24 | 1.8 | 0.96 |
| BEST1 | | 109.5 | 0.9 | 4.42 | 1.38 | | 112.9 | 9.83 | 1.21 | 0.64 |
| BEST2 | | 103.3 | 4.5 | 0.72 | 0.34 | | 90.2 | 4.12 | -1.35 | -0.44 |
| BEST3 | | 104.1 | 14.89 | 0.27 | 0.15 | | 133.6 | 17.56 | 1.74 | 0.81 |

| | | | | | | | | | | |
|---|---|---|---|---|---|---|---|---|---|---|
| BEST4 | | 105.2 | 3.45 | 1.31 | 0.53 | | 113.5 | 17.41 | 0.72 | 0.32 |
| CACHD1 | | 103.7 | 3.58 | 0.98 | 0.44 | | 118.6 | 6.07 | 2.38 | 0.89 |
| CACNA1A | | 112.4 | 3.79 | 2.9 | 1.31 | | 136.5 | 6.32 | 4.83 | 2.02 |
| CACNA1B | | 101.4 | 3.93 | 0.33 | 0.15 | | 96.4 | 19.52 | -0.18 | -0.1 |
| CACNA1C | | 106.9 | 5.17 | 1.23 | 0.55 | | 112.5 | 16.11 | 0.72 | 0.32 |
| CACNA1D | | 102.8 | 1 | 1.21 | 0.38 | | 110.1 | 6.14 | 1.12 | 0.39 |
| CACNA1E | | 99.8 | 2.43 | -0.09 | -0.04 | | 112.5 | 17.22 | 0.68 | 0.3 |
| CACNA1G | | 113.8 | 3.46 | 3.67 | 1.21 | | 137.1 | 8.13 | 3.99 | 1.27 |
| CACNA1H | | 105.6 | 11.61 | 0.46 | 0.25 | | 103.2 | 16.73 | 0.17 | 0.08 |
| CACNA1I | | 102.9 | 5.24 | 0.52 | 0.23 | | 102.2 | 9.66 | 0.19 | 0.07 |
| CACNA1S | | 100.6 | 2.63 | 0.19 | 0.07 | | 119.6 | 8.64 | 1.8 | 0.68 |
| CACNA2D4 | | 101.6 | 8.82 | 0.18 | 0.09 | | 87.2 | 4.86 | -1.66 | -0.56 |
| CACNB1 | | 115.8 | 3.32 | 4.09 | 1.77 | | 132.8 | 18.9 | 1.7 | 0.92 |
| CACNB2 | | 85.4 | 14.84 | -0.98 | -0.49 | | 69.2 | 9.88 | -2.67 | -1.08 |
| CACNB3 | | 103.5 | 6.63 | 0.52 | 0.25 | | 96.7 | 6.72 | -0.37 | -0.14 |
| CACNB4 | | 106 | 4.7 | 1.18 | 0.57 | | 111.4 | 14.96 | 0.73 | 0.39 |
| CACNG2 | | 94.3 | 2.76 | -1.97 | -0.91 | | 83.1 | 13.52 | -1.12 | -0.47 |
| CACNG3 | | 97.7 | 4.65 | -0.49 | -0.23 | | 88.8 | 7.52 | -1.17 | -0.44 |
| CACNG4 | | 112.8 | 1.66 | 5 | 1.74 | | 120.1 | 5.55 | 2.89 | 1.16 |
| CACNG5 | | 109.7 | 3.73 | 2.3 | 1.04 | | 118.6 | 1.87 | 4.1 | 1.27 |
| CACNG7 | | 104.5 | 1.94 | 1.64 | 0.6 | | 131.2 | 23.04 | 1.33 | 0.74 |
| CATSPER2 | | 102.6 | 2.15 | 1.04 | 0.43 | | 107.1 | 12.88 | 0.51 | 0.23 |
| CATSPER3 | | 103.7 | 3.28 | 1.01 | 0.47 | | 99.8 | 4.62 | -0.03 | -0.01 |
| CHRNA1 | | 98.8 | 3.12 | -0.36 | -0.15 | | 110 | 8.48 | 1.07 | 0.46 |
| CHRNA10 | | 105.3 | 2.55 | 1.78 | 0.59 | | 126.4 | 8.26 | 2.81 | 0.93 |
| CHRNA2 | | 107.6 | 4.6 | 1.53 | 0.73 | | 124.3 | 17.77 | 1.33 | 0.72 |
| CHRNA3 | | 103.9 | 10.65 | 0.35 | 0.18 | | 107.5 | 7.37 | 0.68 | 0.24 |
| CHRNA4 | | 93.5 | 3.68 | -1.71 | -0.8 | | 81.8 | 5.25 | -2.3 | -0.79 |
| CHRNA5 | | 95.8 | 5.59 | -0.72 | -0.34 | | 95.3 | 9.3 | -0.46 | -0.2 |
| CHRNA6 | | 110.3 | 5.14 | 1.86 | 0.83 | | 126.3 | 10.49 | 2.12 | 0.85 |
| CHRNA7 | | 111.3 | 4 | 2.54 | 1.17 | | 115.5 | 14.58 | 1.02 | 0.53 |
| CHRNA9 | | 110.4 | 1.14 | 4.61 | 1.48 | | 111.7 | 7.77 | 1.33 | 0.6 |
| CHRNB1 | | 110.3 | 2.3 | 3.4 | 1.3 | | 124.2 | 20.22 | 1.17 | 0.64 |
| CHRNB2 | | 88.6 | 0.73 | -5.45 | -1.66 | | 110.7 | 3.34 | 2 | 0.7 |
| CHRNB3 | | 104.7 | 4.63 | 0.92 | 0.4 | | 120.4 | 13.26 | 1.38 | 0.58 |
| CHRNB4 | | 105 | 1.79 | 2.13 | 0.63 | | 119.7 | 10.11 | 1.78 | 0.58 |
| CHRNG | | 97.3 | 11.47 | -0.23 | -0.14 | | 121.1 | 24.95 | 0.8 | 0.47 |
| CLCA1 | | 95.3 | 4.05 | -1.09 | -0.49 | | 82.5 | 20.98 | -0.82 | -0.4 |
| CLCA2 | | 92.1 | 3.6 | -2.01 | -0.88 | | 98.5 | 3.39 | -0.29 | -0.1 |
| CLCA4 | | 105.1 | 4.39 | 1.06 | 0.46 | | 115.5 | 11.69 | 1.16 | 0.47 |
| CLCN2 | | 94.8 | 3.95 | -1.18 | -0.5 | | 93.7 | 8.5 | -0.59 | -0.22 |

| | | | | | | | | | | |
|---|---|---|---|---|---|---|---|---|---|---|
| CLCN3 | | 108.1 | 8.98 | 0.9 | 0.44 | | 97.5 | 12.2 | -0.18 | -0.08 |
| CLCN4 | | 107.5 | 5.03 | 1.42 | 0.48 | | 127 | 18.9 | 1.39 | 0.48 |
| CLCN6 | | 100.5 | 3.06 | 0.14 | 0.06 | | 99.9 | 9.79 | -0.01 | 0 |
| CLCNKA | | 100.7 | 5.16 | 0.13 | 0.06 | | 79.9 | 11.42 | -1.56 | -0.66 |
| CLCNKB | | 128 | 29.62 | 0.94 | 0.53 | | 122.9 | 15.02 | 1.34 | 0.6 |
| CLIC1 | | 100.1 | 4.02 | 0.02 | 0.01 | | 86.4 | 8.64 | -1.29 | -0.5 |
| CLIC2 | | 102 | 3.7 | 0.49 | 0.24 | | 117.1 | 14.86 | 1.11 | 0.58 |
| CLIC3 | | 102.9 | 3.21 | 0.8 | 0.37 | | 109.4 | 14.05 | 0.64 | 0.33 |
| CLIC4 | | 99.2 | 3.46 | -0.2 | -0.1 | | 116.6 | 9.95 | 1.54 | 0.74 |
| CLIC5 | | 99.1 | 4.12 | -0.2 | -0.1 | | 113.8 | 9.52 | 1.33 | 0.63 |
| CLIC6 | | 102.6 | 2.48 | 0.66 | 0.23 | | 95.4 | 1.5 | -0.56 | -0.16 |
| CNGA1 | | 96.2 | 1 | -2.86 | -1.05 | | 103.2 | 22.13 | 0.14 | 0.06 |
| CNGA2 | | 95.1 | 6.21 | -0.77 | -0.41 | | 78.7 | 16.4 | -1.26 | -0.67 |
| CNGA3 | | 103.9 | 4.08 | 0.87 | 0.42 | | 128.1 | 11.57 | 2.25 | 1.09 |
| CNGA4 | | 105.1 | 5.79 | 0.86 | 0.42 | | 87.9 | 2.72 | -1.85 | -0.57 |
| CNGB1 | | 102.7 | 6.02 | 0.45 | 0.22 | | 93.8 | 11.2 | -0.49 | -0.2 |
| CNGB3 | | 112.8 | 5.78 | 2.1 | 1.07 | | 104 | 4.33 | 0.69 | 0.26 |
| FXYD1 | | 111.4 | 3.68 | 2.76 | 1.25 | | 120.6 | 6.07 | 2.87 | 1.22 |
| FXYD3 | | 116.7 | 0.28 | 8.81 | 2.57 | | 130.5 | 15.69 | 1.89 | 1.18 |
| FXYD3 | | 108 | 5.03 | 1.52 | 0.52 | | 114.1 | 7.02 | 1.69 | 0.53 |
| FXYD4 | | 106.3 | 5.96 | 1.03 | 0.5 | | 88.9 | 6.3 | -1.29 | -0.46 |
| FXYD6 | | 104.1 | 6.52 | 0.6 | 0.32 | | 110.4 | 3.9 | 1.71 | 0.59 |
| FXYD7 | | 116.2 | 3.1 | 4.46 | 1.92 | | 91.9 | 14.9 | -0.53 | -0.28 |
| GABRA1 | | 93.8 | 11.31 | -0.54 | -0.27 | | 94.7 | 21.83 | -0.23 | -0.11 |
| GABRA2 | | 105.2 | 5.03 | 0.98 | 0.48 | | 117.1 | 18.43 | 0.91 | 0.49 |
| GABRA3 | | 112.5 | 4.16 | 2.8 | 1.38 | | 133.4 | 6.05 | 4.56 | 1.88 |
| GABRA4 | | 105.2 | 3.93 | 1.18 | 0.49 | | 118.4 | 10.8 | 1.45 | 0.59 |
| GABRA5 | | 94.4 | 3.18 | -1.69 | -0.79 | | 85.1 | 4.64 | -1.79 | -0.58 |
| GABRA6 | | 103.7 | 5.2 | 0.66 | 0.29 | | 123.6 | 6.07 | 2.63 | 0.91 |
| GABRB1 | | 105.9 | 3.96 | 1.32 | 0.55 | | 106.4 | 14.04 | 0.41 | 0.18 |
| GABRB2 | | 102.9 | 5.79 | 0.48 | 0.26 | | 124.9 | 6.51 | 3.22 | 1.36 |
| GABRB3 | | 102.8 | 1.93 | 0.98 | 0.34 | | 110 | 10.82 | 0.79 | 0.32 |
| GABRD | | 99.9 | 3.35 | -0.02 | -0.01 | | 111.5 | 12.42 | 0.82 | 0.34 |
| GABRE | | 100.4 | 4.65 | 0.09 | 0.04 | | 86.7 | 4.17 | -1.83 | -0.6 |
| GABRG1 | | 106.8 | 1.78 | 2.87 | 1.09 | | 132.4 | 14.36 | 2.17 | 1.13 |
| GABRG2 | | 105 | 5.05 | 0.98 | 0.47 | | 96.5 | 5.25 | -0.44 | -0.15 |
| GABRG3 | | 95.8 | 2.68 | -1.49 | -0.69 | | 96.1 | 6.5 | -0.42 | -0.15 |
| GABRP | | 93.1 | 0.3 | -4.36 | -1.27 | | 81.4 | 16.9 | -1.07 | -0.57 |
| GABRQ | | 101.1 | 6.98 | 0.15 | 0.08 | | 92.8 | 7.05 | -0.78 | -0.29 |
| GABRR1 | | 95 | 6.47 | -0.76 | -0.4 | | 90.9 | 9.24 | -0.9 | -0.42 |
| GABRR2 | | 104.2 | 6.23 | 0.65 | 0.34 | | 104.8 | 8.81 | 0.48 | 0.22 |

| | | | | | | | | | | |
|---|---|---|---|---|---|---|---|---|---|---|
| GABRR3 | | 101.8 | 3.09 | 0.56 | 0.25 | | 113 | 7.77 | 1.42 | 0.57 |
| GLRA1 | | 107.2 | 2.33 | 2.58 | 1.08 | | 110.8 | 1.37 | 2.56 | 0.77 |
| GLRA2 | | 107.9 | 5.07 | 1.52 | 0.72 | | 125.9 | 6.1 | 3.31 | 1.24 |
| GLRA3 | | 105.7 | 5.64 | 0.99 | 0.48 | | 100.5 | 7.8 | 0.05 | 0.02 |
| GLRB | | 99.1 | 3 | -0.26 | -0.12 | | 105.1 | 8.42 | 0.53 | 0.21 |
| GRIA1 | | 96.4 | 2.31 | -1.43 | -0.62 | | 93.3 | 9.12 | -0.61 | -0.24 |
| GRIA2 | | 114.2 | 2.66 | 4.37 | 1.78 | | 133.1 | 17.92 | 1.8 | 0.98 |
| GRIA3 | | 96.2 | 5.16 | -0.71 | -0.37 | | 103.2 | 2.29 | 0.69 | 0.22 |
| GRIA4 | | 93.9 | 17.32 | -0.35 | -0.2 | | 93.8 | 27.59 | -0.22 | -0.12 |
| GRID1 | | 116 | 0.41 | 8.31 | 2.44 | | 105 | 5.98 | 0.71 | 0.3 |
| GRID2 | | 106 | 2.82 | 1.71 | 0.66 | | 114.6 | 14.32 | 0.93 | 0.4 |
| GRIK1 | | 102.6 | 2.34 | 0.84 | 0.31 | | 112.5 | 9.94 | 1.04 | 0.41 |
| GRIK2 | | 109.3 | 4.78 | 1.8 | 0.79 | | 117 | 1.56 | 2.5 | 0.73 |
| GRIK3 | | 113.4 | 4.74 | 2.63 | 1.28 | | 136.3 | 14.68 | 2.39 | 1.27 |
| GRIK4 | | 104.4 | 9.88 | 0.44 | 0.24 | | 99.8 | 4.98 | -0.02 | -0.01 |
| GRIK5 | | 113 | 1.95 | 5.19 | 2.03 | | 137.9 | 16.38 | 2.24 | 1.19 |
| GRIN1 | | 107.8 | 3.9 | 1.84 | 0.89 | | 106.8 | 19.94 | 0.33 | 0.18 |
| GRIN2A | | 108.4 | 2.86 | 2.59 | 1.25 | | 127.4 | 3.77 | 4.88 | 1.78 |
| GRIN2B | | 97.4 | 5.3 | -0.46 | -0.21 | | 93.4 | 9.93 | -0.55 | -0.22 |
| GRIN2C | | 107.2 | 4.48 | 1.46 | 0.63 | | 122.1 | 6.03 | 2.47 | 0.85 |
| GRIN2D | | 103.5 | 11.07 | 0.31 | 0.17 | | 122.9 | 15.1 | 1.45 | 0.75 |
| GRIN3A | | 120.6 | 25.25 | 0.82 | 0.47 | | 108.1 | 8.34 | 0.84 | 0.37 |
| GRIN3B | | 102.1 | 8.81 | 0.23 | 0.13 | | 112.3 | 9.04 | 1.21 | 0.55 |
| HCN1 | | 102 | 3.72 | 0.49 | 0.23 | | 103.9 | 22.59 | 0.17 | 0.09 |
| HCN2 | | 102 | 13.62 | 0.14 | 0.08 | | 104 | 15.78 | 0.23 | 0.1 |
| HCN3 | | 94.9 | 4.16 | -1.15 | -0.51 | | 109.8 | 18.14 | 0.54 | 0.26 |
| HCN4 | | 106.5 | 9.5 | 0.68 | 0.37 | | 109.6 | 7.81 | 1.06 | 0.46 |
| HTR3A | | 92.4 | 4.55 | -1.57 | -0.71 | | 105.9 | 10.78 | 0.53 | 0.25 |
| HTR3B | | 96.8 | 3.19 | -0.89 | -0.38 | | 112.6 | 9.77 | 1.24 | 0.58 |
| HTR3C | | 93.7 | 2.36 | -2.2 | -0.86 | | 93.1 | 8.93 | -0.74 | -0.34 |
| HTR3D | | 109.1 | 4.83 | 1.83 | 0.86 | | 125.1 | 14.15 | 1.67 | 0.76 |
| HTR3E | | 91.2 | 6.58 | -1.29 | -0.61 | | 93.1 | 6.25 | -1.01 | -0.44 |
| ITPR1 | | 105.1 | 1.1 | 2.64 | 0.88 | | 103.4 | 8.8 | 0.35 | 0.16 |
| ITPR2 | | 104.7 | 3.34 | 1.28 | 0.6 | | 118.4 | 13.6 | 1.3 | 0.67 |
| ITPR3 | | 112.5 | 2.43 | 4.08 | 1.62 | | 128.2 | 7.33 | 3.41 | 1.54 |
| KCNA1 | | 113.4 | 4.1 | 2.97 | 1.39 | | 99.8 | 22.44 | -0.01 | -0.01 |
| KCNA10 | | 75.4 | 17.81 | -1.37 | -0.67 | | 77.2 | 18.24 | -1.17 | -0.53 |
| KCNA2 | | 104.3 | 3.13 | 1.08 | 0.4 | | 103.5 | 9.89 | 0.29 | 0.11 |
| KCNA4 | | 93.3 | 10.13 | -0.64 | -0.3 | | 86.1 | 14.23 | -0.88 | -0.38 |
| KCNA5 | | 107.4 | 11.05 | 0.65 | 0.34 | | 113.9 | 15.93 | 0.78 | 0.35 |
| KCNA6 | | 106.1 | 6.79 | 0.87 | 0.46 | | 122.1 | 2.91 | 4.01 | 1.3 |

| | | | | | | | | | | |
|---|---|---|---|---|---|---|---|---|---|---|
| KCNA7 | | 120 | 19.3 | 1.03 | 0.57 | | 115.7 | 26.13 | 0.57 | 0.29 |
| KCNAB1 | | 111.3 | 5.88 | 1.82 | 0.93 | | 134.5 | 20.65 | 1.64 | 0.9 |
| KCNAB2 | | 112 | 6.1 | 1.87 | 0.96 | | 123.8 | 15.8 | 1.47 | 0.78 |
| KCNAB3 | | 99.7 | 6.5 | -0.04 | -0.02 | | 100 | 9.06 | 0 | 0 |
| KCNB1 | | 111 | 7.48 | 1.42 | 0.76 | | 109.3 | 8.55 | 0.99 | 0.47 |
| KCNB2 | | 111.9 | 3.36 | 3.2 | 1.49 | | 123.3 | 3.46 | 4.31 | 1.49 |
| KCNC1 | | 108.8 | 4.53 | 1.84 | 0.92 | | 112 | 13.98 | 0.83 | 0.43 |
| KCNC2 | | 93.1 | 6.73 | -1 | -0.53 | | 120.5 | 4.57 | 3.13 | 1.14 |
| KCNC4 | | 100.6 | 3.29 | 0.16 | 0.07 | | 100 | 9.59 | 0 | 0 |
| KCND1 | | 110.2 | 2.13 | 3.85 | 1.55 | | 141.6 | 8.48 | 4.4 | 2.02 |
| KCND2 | | 103.2 | 2.74 | 1.02 | 0.44 | | 125.3 | 15.47 | 1.58 | 0.83 |
| KCND3 | | 90.8 | 10.7 | -0.84 | -0.4 | | 101.8 | 14.31 | 0.11 | 0.05 |
| KCNE1 | | 125.4 | 36.08 | 0.7 | 0.41 | | 128.3 | 29.88 | 0.94 | 0.52 |
| KCNE1L | | 126.9 | 26.39 | 1.02 | 0.58 | | 132.6 | 17.22 | 1.83 | 0.96 |
| KCNE2 | | 95.6 | 1.87 | -1.81 | -0.67 | | 94.7 | 5.71 | -0.76 | -0.29 |
| KCNE3 | | 103 | 3.69 | 0.78 | 0.37 | | 84.3 | 9.28 | -1.36 | -0.52 |
| KCNF1 | | 91.8 | 2.11 | -3.09 | -1.17 | | 105.9 | 11.45 | 0.5 | 0.24 |
| KCNG1 | | 97.9 | 2.76 | -0.65 | -0.27 | | 102.6 | 6.91 | 0.35 | 0.15 |
| KCNG2 | | 111.3 | 8.17 | 1.36 | 0.74 | | 130.7 | 7.26 | 3.55 | 1.49 |
| KCNG3 | | 95.4 | 4.12 | -1.03 | -0.46 | | 94 | 9.68 | -0.59 | -0.28 |
| KCNG4 | | 105 | 2.38 | 1.75 | 0.73 | | 119.6 | 6.35 | 2.58 | 1.08 |
| KCNH1 | | 95 | 3.23 | -1.51 | -0.71 | | 86.6 | 16.1 | -0.76 | -0.33 |
| KCNH2 | | 105.4 | 13.91 | 0.38 | 0.2 | | 101.2 | 5.46 | 0.12 | 0.04 |
| KCNH3 | | 121.4 | 4.21 | 4.64 | 2.19 | | 111.1 | 5.17 | 1.73 | 0.7 |
| KCNH4 | | 96.8 | 4.37 | -0.69 | -0.31 | | 85 | 11.32 | -1.29 | -0.61 |
| KCNH5 | | 97.6 | 1.67 | -1.28 | -0.54 | | 114.5 | 19.7 | 0.7 | 0.32 |
| KCNH6 | | 122.8 | 8.51 | 2.62 | 1.42 | | 151.8 | 30.12 | 1.71 | 0.96 |
| KCNH7 | | 101 | 2.85 | 0.32 | 0.14 | | 117.9 | 11.22 | 1.46 | 0.64 |
| KCNH8 | | 95.4 | 2.99 | -1.43 | -0.63 | | 111.7 | 14.1 | 0.78 | 0.36 |
| KCNJ1 | | 86.3 | 10.8 | -1.27 | -0.63 | | 87 | 14.88 | -0.79 | -0.34 |
| KCNJ10 | | 108.5 | 2.37 | 2.82 | 1.11 | | 111.2 | 5.12 | 1.75 | 0.7 |
| KCNJ11 | | 108.9 | 5.36 | 1.56 | 0.78 | | 79.5 | 38.71 | -0.53 | -0.3 |
| KCNJ13 | | 94.6 | 3.96 | -1.26 | -0.56 | | 76.7 | 7.88 | -2.79 | -1.26 |
| KCNJ14 | | 118.1 | 0.89 | 8.71 | 2.71 | | 137.2 | 25.39 | 1.45 | 0.81 |
| KCNJ15 | | 103.9 | 6.89 | 0.56 | 0.27 | | 115.6 | 6.54 | 1.72 | 0.61 |
| KCNJ16 | | 92.2 | 9.31 | -0.81 | -0.38 | | 79.3 | 12.56 | -1.44 | -0.6 |
| KCNJ2 | | 102.6 | 3.48 | 0.69 | 0.3 | | 95.8 | 3.77 | -0.57 | -0.18 |
| KCNJ3 | | 102.5 | 3.83 | 0.62 | 0.28 | | 87.4 | 7.06 | -1.34 | -0.49 |
| KCNJ4 | | 103.6 | 4.9 | 0.7 | 0.32 | | 97.9 | 18.32 | -0.11 | -0.05 |
| KCNJ5 | | 103.4 | 5.11 | 0.65 | 0.3 | | 92.7 | 11.64 | -0.56 | -0.23 |
| KCNJ6 | | 96.9 | 3.62 | -0.71 | -0.28 | | 103.1 | 13.48 | 0.21 | 0.09 |

| | | | | | | | | | | |
|---|---|---|---|---|---|---|---|---|---|---|
| KCNJ8 | | 113.1 | 3.58 | 3.44 | 1.55 | | 134.5 | 18.06 | 1.84 | 0.86 |
| KCNJ9 | | 100.3 | 10.02 | 0.03 | 0.01 | | 101.6 | 20.78 | 0.08 | 0.04 |
| KCNK1 | | 102.2 | 8.78 | 0.25 | 0.12 | | 114 | 14.25 | 0.93 | 0.42 |
| KCNK10 | | 108.3 | 9.48 | 0.87 | 0.43 | | 117.9 | 30 | 0.59 | 0.29 |
| KCNK12 | | 105.8 | 6.48 | 0.87 | 0.46 | | 117.2 | 5.79 | 2.31 | 0.9 |
| KCNK13 | | 104.8 | 10.63 | 0.43 | 0.23 | | 94.9 | 13.88 | -0.32 | -0.14 |
| KCNK15 | | 102.6 | 6.46 | 0.39 | 0.19 | | 109.8 | 5.15 | 1.37 | 0.49 |
| KCNK16 | | 100.4 | 8.2 | 0.04 | 0.02 | | 93.4 | 13.78 | -0.43 | -0.19 |
| KCNK17 | | 78.4 | 17.72 | -1.21 | -0.59 | | 77.5 | 26.02 | -0.84 | -0.39 |
| KCNK18 | | 99.3 | 6.17 | -0.11 | -0.05 | | 110.8 | 9.07 | 0.95 | 0.36 |
| KCNK2 | | 95.3 | 2.96 | -1.53 | -0.71 | | 96.7 | 9.03 | -0.29 | -0.11 |
| KCNK4 | | 121.9 | 18.69 | 1.17 | 0.58 | | 113 | 21.36 | 0.58 | 0.27 |
| KCNK5 | | 90.5 | 8.2 | -1.1 | -0.51 | | 88.4 | 13.54 | -0.77 | -0.32 |
| KCNK6 | | 131.2 | 28.92 | 1.08 | 0.62 | | 137.7 | 14.28 | 2.51 | 1.28 |
| KCNK7 | | 104.1 | 4.58 | 0.84 | 0.42 | | 103.3 | 17.46 | 0.19 | 0.1 |
| KCNK9 | | 96.8 | 2.35 | -1.15 | -0.46 | | 98 | 5.95 | -0.23 | -0.08 |
| KCNMA1 | | 97.4 | 3.88 | -0.62 | -0.27 | | 91.3 | 10.6 | -0.8 | -0.38 |
| KCNMB1 | | 96.6 | 2.35 | -1.2 | -0.47 | | 101.4 | 7.78 | 0.17 | 0.08 |
| KCNMB2 | | 84.5 | 12.95 | -1.17 | -0.56 | | 46.2 | 23.33 | -2.29 | -1.12 |
| KCNMB3 | | 88.2 | 5.44 | -2.09 | -0.97 | | 88.2 | 6.79 | -1.61 | -0.71 |
| KCNMB4 | | 101.5 | 5.47 | 0.27 | 0.13 | | 108.9 | 9.66 | 0.77 | 0.3 |
| KCNN1 | | 102.5 | 3.29 | 0.71 | 0.32 | | 117.7 | 10.11 | 1.58 | 0.67 |
| KCNN2 | | 96.7 | 6.25 | -0.48 | -0.21 | | 95.1 | 5.06 | -0.83 | -0.33 |
| KCNN3 | | 94.5 | 3.26 | -1.53 | -0.65 | | 98.6 | 12.23 | -0.11 | -0.05 |
| KCNN4 | | 103.5 | 9.67 | 0.36 | 0.17 | | 118.2 | 29.36 | 0.62 | 0.35 |
| KCNQ1 | | 102.5 | 3.39 | 0.69 | 0.3 | | 98.5 | 5.1 | -0.18 | -0.06 |
| KCNQ2 | | 98.1 | 2.7 | -0.51 | -0.18 | | 95.5 | 8.21 | -0.51 | -0.23 |
| KCNQ3 | | 93.5 | 5.79 | -1.03 | -0.45 | | 97.9 | 12.17 | -0.15 | -0.06 |
| KCNQ4 | | 95.6 | 23.96 | -0.18 | -0.11 | | 102.1 | 35.16 | 0.06 | 0.03 |
| KCNQ5 | | 101.3 | 7.16 | 0.18 | 0.09 | | 101.7 | 3.33 | 0.29 | 0.1 |
| KCNT1 | | 112.8 | 6.17 | 2.04 | 0.98 | | 111.4 | 11.44 | 0.91 | 0.4 |
| KCNT2 | | 96.3 | 4.99 | -0.72 | -0.34 | | 106.7 | 6.45 | 0.82 | 0.31 |
| KCNV1 | | 114.5 | 8.98 | 1.6 | 0.79 | | 131.8 | 16.75 | 1.82 | 0.84 |
| KCNV2 | | 99 | 4.04 | -0.2 | -0.08 | | 88 | 8.17 | -1.13 | -0.42 |
| KCTD17 | | 107.4 | 5.72 | 1.26 | 0.68 | | 146.2 | 29.97 | 1.52 | 0.85 |
| KCTD2 | | 72.7 | 18.29 | -1.49 | -0.85 | | 82.8 | 9.45 | -1.63 | -0.75 |
| KCTD3 | | 100.9 | 6.55 | 0.14 | 0.07 | | 114.6 | 21.49 | 0.66 | 0.32 |
| KCTD5 | | 124.8 | 19.46 | 1.27 | 0.88 | | 174.5 | 3.35 | 12.94 | 4.41 |
| LENG9 | | 104.1 | 3.27 | 1.1 | 0.45 | | 107.3 | 7.29 | 0.57 | 0.18 |
| MCOLN1 | | 106.4 | 6.43 | 0.97 | 0.47 | | 131.8 | 30.55 | 1.03 | 0.5 |
| MCOLN2 | | 94.3 | 7.93 | -0.68 | -0.31 | | 91.6 | 16.43 | -0.5 | -0.24 |

| | | | | | | | | | | |
|---|---|---|---|---|---|---|---|---|---|---|
| MCOLN3 | | 105.2 | 5.64 | 0.9 | 0.43 | | 129.6 | 21.89 | 1.32 | 0.63 |
| NALCN | | 97.6 | 9.74 | -0.23 | -0.11 | | 85.4 | 12.29 | -1.15 | -0.55 |
| NUPL2 | | 98.6 | 4.27 | -0.28 | -0.11 | | 108.4 | 9.31 | 0.72 | 0.28 |
| P2RX1 | | 87.5 | 7.72 | -1.54 | -0.71 | | 89.4 | 14.17 | -0.68 | -0.29 |
| P2RX2 | | 102.1 | 8.46 | 0.25 | 0.12 | | 80.9 | 12.43 | -1.37 | -0.58 |
| P2RX3 | | 95 | 4.45 | -0.96 | -0.39 | | 88.4 | 10.23 | -1.09 | -0.51 |
| P2RX4 | | 87.3 | 9.02 | -1.34 | -0.62 | | 95.9 | 9.91 | -0.4 | -0.18 |
| P2RX6 | | 95.8 | 7.49 | -0.53 | -0.24 | | 87.4 | 7.31 | -1.59 | -0.7 |
| P2RX7 | | 105.9 | 8.5 | 0.66 | 0.31 | | 91.9 | 10.32 | -0.66 | -0.26 |
| PKD2 | | 111.9 | 4.02 | 2.71 | 1.17 | | 117.1 | 6.66 | 1.38 | 0.44 |
| PKD2L1 | | 103.9 | 3.98 | 0.93 | 0.42 | | 98.6 | 6.32 | -0.16 | -0.06 |
| PKD2L2 | | 98.1 | 2.13 | -0.78 | -0.34 | | 109.5 | 2.09 | 1.78 | 0.54 |
| PKDREJ | | 112.9 | 2.12 | 4.64 | 1.71 | | 110.1 | 9.64 | 0.7 | 0.24 |
| RYR1 | | 106.2 | 7.48 | 0.81 | 0.39 | | 111.8 | 12.22 | 0.86 | 0.36 |
| RYR2 | | 101 | 2.51 | 0.32 | 0.13 | | 98.8 | 11.21 | -0.1 | -0.05 |
| RYR3 | | 105.8 | 5.25 | 1.07 | 0.5 | | 106.9 | 4.84 | 0.87 | 0.29 |
| SCN10A | | 104.9 | 2.83 | 1.6 | 0.7 | | 124.6 | 11.62 | 1.95 | 0.85 |
| SCN11A | | 93.7 | 3.58 | -1.61 | -0.7 | | 102.2 | 5.79 | 0.34 | 0.14 |
| SCN1A | | 100.5 | 6.9 | 0.07 | 0.03 | | 98.1 | 7.88 | -0.22 | -0.1 |
| SCN1B | | 99.9 | 4.43 | -0.02 | -0.01 | | 127 | 15.13 | 1.7 | 0.78 |
| SCN2A | | 113.2 | 7.82 | 1.59 | 0.8 | | 103.8 | 2.79 | 0.93 | 0.33 |
| SCN2B | | 99.5 | 4.41 | -0.11 | -0.05 | | 95.4 | 11.77 | -0.35 | -0.14 |
| SCN3A | | 102.4 | 4.26 | 0.54 | 0.25 | | 119.7 | 14.39 | 1.3 | 0.59 |
| SCN3B | | 112.9 | 4.38 | 2.82 | 1.31 | | 127.9 | 12.79 | 2.03 | 0.9 |
| SCN4A | | 97 | 4.66 | -0.61 | -0.28 | | 115.5 | 9.51 | 1.57 | 0.73 |
| SCN4B | | 117.2 | 6.97 | 2.42 | 1.17 | | 138 | 16.32 | 2.23 | 1.03 |
| SCN5A | | 85.9 | 6.1 | -2.23 | -1.05 | | 95.1 | 13.27 | -0.36 | -0.17 |
| SCN7A | | 89.8 | 11.93 | -0.83 | -0.4 | | 65.2 | 20.76 | -1.66 | -0.81 |
| SCN8A | | 91.3 | 1.78 | -2.66 | -0.86 | | 94.5 | 1.61 | -1.59 | -0.5 |
| SCN9A | | 114.2 | 3.92 | 3.29 | 1.41 | | 121.7 | 4.84 | 1.87 | 0.58 |
| SCNN1A | | 91.7 | 7.42 | -1.04 | -0.47 | | 79.7 | 8.52 | -2.24 | -1.01 |
| SCNN1B | | 111.6 | 10.89 | 1.06 | 0.52 | | 136.6 | 27.97 | 1.29 | 0.63 |
| SCNN1D | | 105.5 | 7.9 | 0.68 | 0.33 | | 137.5 | 29.76 | 1.24 | 0.61 |
| SCNN1G | | 96.2 | 0.63 | -2.18 | -0.66 | | 108.9 | 23.43 | 0.38 | 0.21 |
| SHKBP1 | | 98.7 | 2.63 | -0.46 | -0.2 | | 103.6 | 6.56 | 0.44 | 0.17 |
| SHROOM1 | | 93.3 | 7.15 | -0.88 | -0.4 | | 78.2 | 9.08 | -1.92 | -0.73 |
| SHROOM2 | | 110.9 | 7.04 | 1.52 | 0.73 | | 156.7 | 50.85 | 1.11 | 0.55 |
| SHROOM4 | | 122.6 | 5.49 | 3.9 | 1.98 | | 120.3 | 9 | 1.47 | 0.51 |
| SLC9C1 | | 92.8 | 3.19 | -2.12 | -0.94 | | 106.1 | 10.49 | 0.52 | 0.22 |
| TAS1R1 | | 102.4 | 7.3 | 0.32 | 0.14 | | 97.7 | 7.58 | -0.22 | -0.08 |
| TAS1R3 | | 98.4 | 7.3 | -0.2 | -0.09 | | 93.8 | 5.1 | -0.73 | -0.24 |

| | | | | | | | | | | |
|---|---|---|---|---|---|---|---|---|---|---|
| TMC1 | | 107.7 | 9.15 | 0.8 | 0.4 | | 98 | 4.28 | -0.21 | -0.07 |
| TMC2 | | 111.6 | 4.24 | 2.52 | 1.1 | | 114.2 | 9.38 | 1.01 | 0.35 |
| TMC3 | | 92.3 | 6.86 | -1.06 | -0.47 | | 87.6 | 8.6 | -1.13 | -0.42 |
| TMC4 | | 104.6 | 11.03 | 0.41 | 0.19 | | 114.3 | 12.64 | 0.99 | 0.41 |
| TMC5 | | 99.6 | 7.86 | -0.04 | -0.02 | | 89 | 3.38 | -2.43 | -0.88 |
| TMC6 | | 108.7 | 5.67 | 1.48 | 0.7 | | 111.1 | 9.14 | 1 | 0.39 |
| TMC7 | | 84.3 | 18.23 | -0.86 | -0.43 | | 91 | 28.2 | -0.31 | -0.15 |
| TMC8 | | 104.8 | 2.49 | 1.57 | 0.6 | | 111.8 | 8.05 | 0.89 | 0.3 |
| TMEM235 | | 90.3 | 4.23 | -1.94 | -0.81 | | 77.4 | 12.58 | -1.75 | -0.94 |
| TPCN1 | | 90.8 | 5.4 | -1.64 | -0.76 | | 68.7 | 31.83 | -0.98 | -0.48 |
| TPCN2 | | 98.5 | 4.67 | -0.3 | -0.13 | | 110.5 | 11.23 | 0.88 | 0.4 |
| TRPA1 | | 99.1 | 6.22 | -0.13 | -0.06 | | 126.5 | 12.21 | 2.06 | 0.94 |
| TRPC1 | | 90.3 | 4.46 | -2.06 | -0.94 | | 96.4 | 15.75 | -0.22 | -0.11 |
| TRPC3 | | 94.3 | 4.85 | -1.12 | -0.52 | | 99.6 | 23.04 | -0.02 | -0.01 |
| TRPC4 | | 96 | 5.17 | -0.68 | -0.28 | | 99.6 | 4.43 | -0.07 | -0.03 |
| TRPC5 | | 98.5 | 1.77 | -0.79 | -0.34 | | 101.3 | 8.1 | 0.13 | 0.05 |
| TRPC6 | | 99.6 | 2.21 | -0.17 | -0.08 | | 93.3 | 6.23 | -0.73 | -0.25 |
| TRPC7 | | 95.9 | 1.28 | -1.34 | -0.41 | | 85.8 | 4.29 | -2.69 | -1.04 |
| TRPM1 | | 106.5 | 11.44 | 0.55 | 0.29 | | 103.7 | 10.21 | 0.28 | 0.11 |
| TRPM2 | | 94 | 6.48 | -0.85 | -0.37 | | 87.2 | 4.38 | -2.41 | -0.94 |
| TRPM3 | | 93.6 | 6.16 | -0.94 | -0.41 | | 90.4 | 9.1 | -1 | -0.46 |
| TRPM4 | | 118 | 17.87 | 1.01 | 0.5 | | 128.1 | 14.86 | 1.74 | 0.76 |
| TRPM5 | | 99.5 | 4.58 | -0.1 | -0.05 | | 110 | 22.04 | 0.43 | 0.2 |
| TRPM6 | | 96.1 | 7.05 | -0.51 | -0.23 | | 92.2 | 9.84 | -0.76 | -0.35 |
| TRPM7 | | 107.8 | 11.42 | 0.66 | 0.35 | | 105.8 | 15.31 | 0.33 | 0.15 |
| TRPM8 | | 102.4 | 2.73 | 0.81 | 0.35 | | 124 | 7.64 | 2.64 | 1.05 |
| TRPV1 | | 105.9 | 3.07 | 1.8 | 0.8 | | 118.7 | 11.63 | 1.48 | 0.65 |
| TRPV2 | | 121.5 | 14.35 | 1.49 | 0.74 | | 114.9 | 15.79 | 0.88 | 0.39 |
| TRPV3 | | 97.3 | 4.15 | -0.65 | -0.31 | | 96.8 | 4.24 | -0.4 | -0.13 |
| TRPV4 | | 114.6 | 13.19 | 1.1 | 0.54 | | 102.7 | 11.13 | 0.21 | 0.09 |
| TRPV5 | | 103.3 | 3.68 | 0.85 | 0.39 | | 121.9 | 7.7 | 2.39 | 0.95 |
| TRPV6 | | 96.4 | 3.77 | -0.92 | -0.44 | | 96.5 | 8.59 | -0.32 | -0.12 |
| TTYH1 | | 97.7 | 6.63 | -0.33 | -0.16 | | 98.2 | 4.9 | -0.29 | -0.11 |
| TTYH2 | | 118.5 | 7.68 | 2.38 | 1.16 | | 153.1 | 39.68 | 1.33 | 0.65 |
| TTYH3 | | 65.1 | 15.07 | -2.3 | -1.14 | | 76.6 | 19.43 | -1.18 | -0.57 |
| VDAC1 | | 105.8 | 12.68 | 0.44 | 0.24 | | 87.9 | 32.5 | -0.36 | -0.19 |
| VDAC2 | | 109.7 | 8.74 | 1.05 | 0.53 | | 99.7 | 4.3 | -0.03 | -0.01 |
| VDAC3 | | 111.5 | 2.63 | 3.58 | 1.4 | | 117.5 | 8.99 | 1.27 | 0.43 |
